# Common High Altitudes Illnesses a Primer for Healthcare Provider

**DOI:** 10.9734/BJMMR/2015/17501

**Published:** 2015-04-17

**Authors:** Vahid Mohsenin

**Affiliations:** 1 Department of Pulmonary, Critical Care and Sleep Medicine Yale School of Medicine, Yale University, New Haven, Connecticut, USA

**Keywords:** High altitude, acute mountain sickness, high altitude pulmonary edema, sleep disturbances

## Abstract

Exposure to high altitude imposes significant strain on cardiopulmonary system and the brain. As a consequence, sojourners to high altitude frequently experience sleep disturbances, often reporting restless and sleepless nights.

At altitudes above 3,000 meters (9,800 ft) almost all healthy subjects develop periodic breathing especially during NREM sleep.

Sleep architecture gradually improves with increased NREM and REM sleep despite persistence of periodic breathing.

The primary reason for periodic breathing at high altitude is a hypoxic-induced increase in chemoreceptor sensitivity to changes in PaCO_2_ – both above and below eupnea, leading to periods of apnea and hyperpnea.

Acetazolamide improves sleep by reducing the periodic breathing through development of metabolic acidosis and induced hyperventilation decreasing the plant gain and widening the PCO_2_ reserve. This widening of the PCO_2_ reserve impedes development of central apneas during sleep. Benzodiazepines and GABA receptor antagonist such as zolpidem improve sleep without affecting breathing pattern or cognitive functions.

## 1. INTRODUCTION

### 1.1 Exposure to High Altitude

Each year several million people worldwide travel from areas of low elevation to altitudes over 2,500 meters (8,000 ft). Empirically, 2,500 meters has been used as the threshold for highaltitude illnesses because of higher risk of development of acute mountain sickness (AMS). With the increasing popularity of mountain sports such as skiing, climbing, and snowshoeing, and the latest trend in adventure travel to places like the Andes and Himalayas, we can expect the incidence of high altitude exposure to continue to grow. These often rapid ascents of non-acclimatized individuals place them at an increased risk for AMS, insomnia and sleep disordered breathing. Primary care providers are frequently being consulted for advice on prevention and treatment of high altitude-related illnesses by their patients planning trips to high altitude destinations. This review will focus on the diagnosis and management of these common disorders of high altitudes. The discussion on less common high altitude illnesses such as high altitude pulmonary edema (HAPE) or high altitude cerebral edema (HACE) is beyond the scope of this reviewed which is well covered by other authors [1].

### 1.2 Physiologic Adjustment to High Altitude

**Fig. 1:**
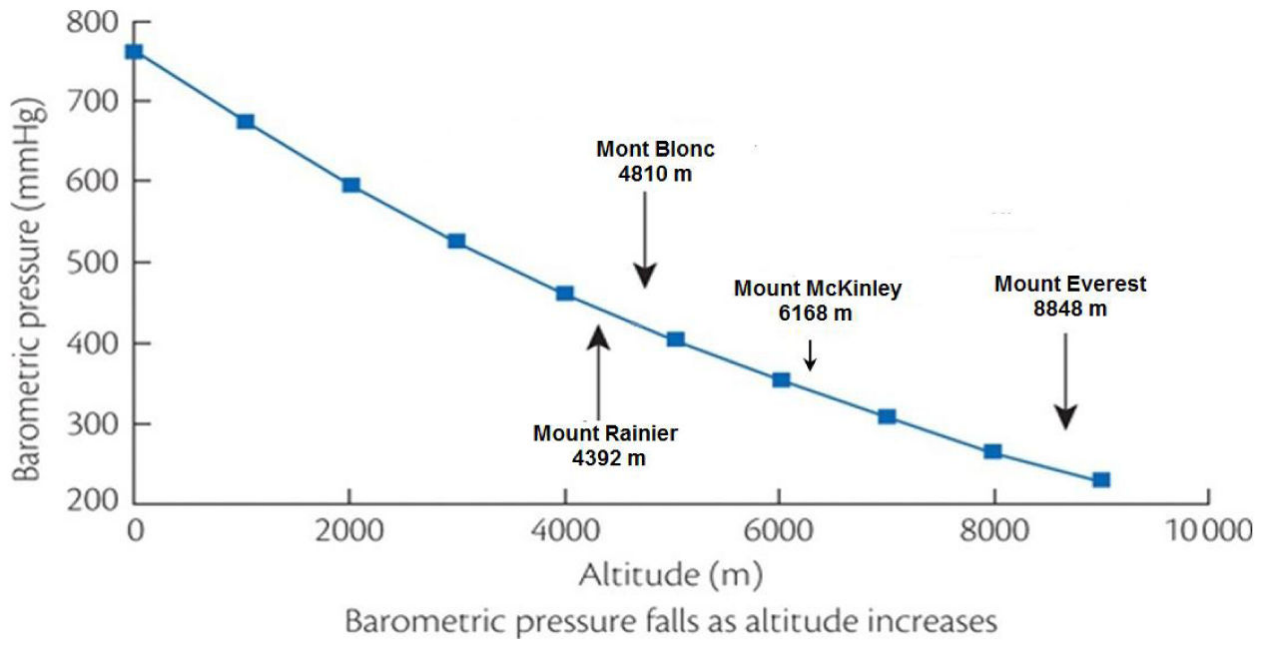
Relationship between altitude and barometric pressure. Inspired oxygen pressures on the summits of Mount Rainier, Mont Blanc France, Mount McKinley (Denali) and Mount Everest are 82, 80, 61 and 43 mmHg, respectively

Primary among the changes in physical environment that attend the ascent to altitude is a decrease in barometric pressure such that although the fractional concentration of O_2_ is similar to that at sea level, O_2_ tension—the product of fractional concentration and barometric pressure—is reduced (Fig. 1). This decreased O_2_ tension of ambient air presents a threat to tissue oxygenation and elicits a series of responses that may act to minimize tissue hypoxia. These consist of increases in ventilation, cardiac output, circulating red cell concentration and adaptive changes in peripheral tissue, including increased spatial density of capillaries and mitochondria.

### 1.3 Acute Mountain Illness

**Fig. 2:**
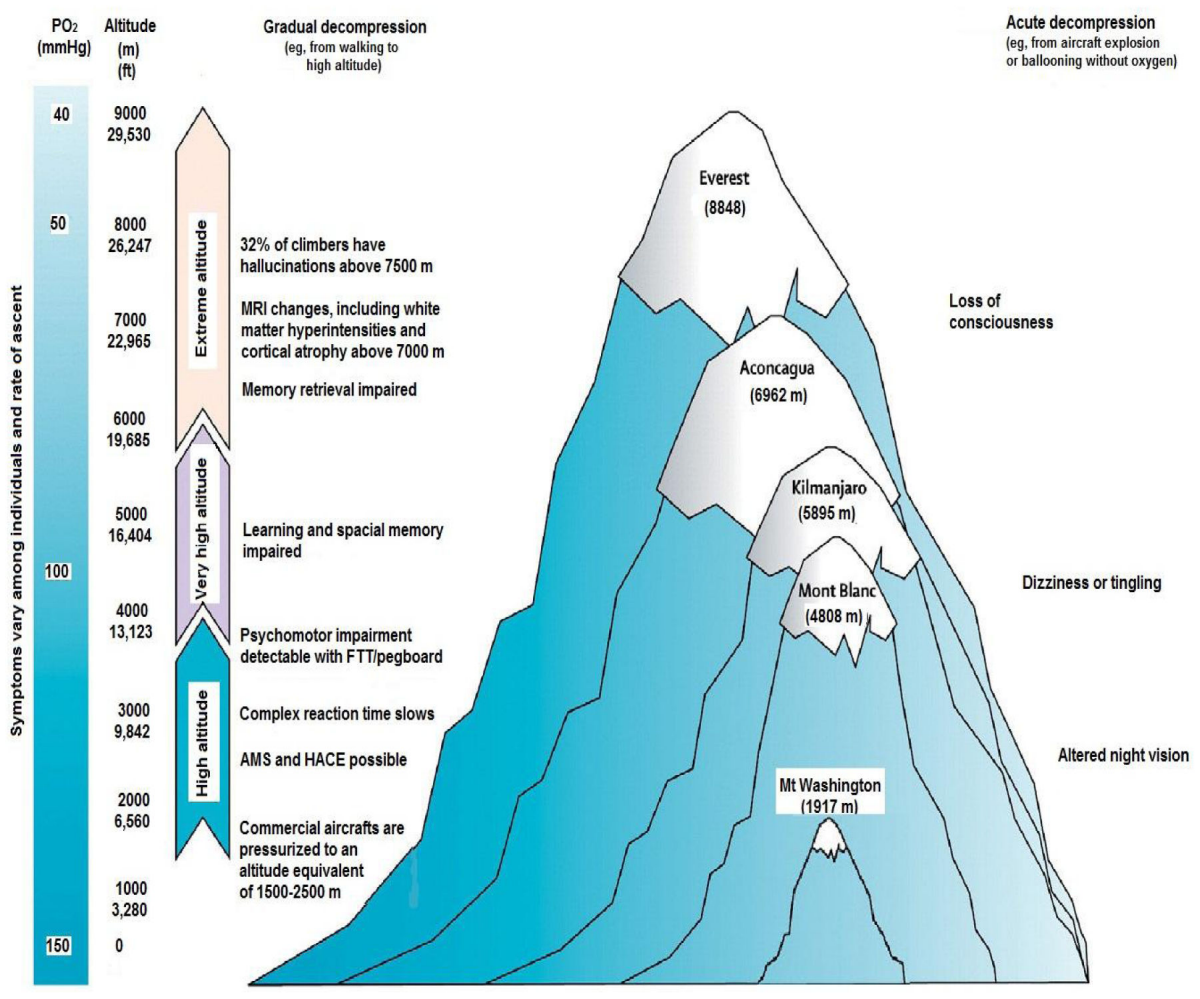
The spectrum of high altitude symptoms and illnesses in relationship to high altitude exposure

AMS is the most common form of altitude illness, affecting, for example, 25% of all visitors sleeping above 2,500 meters (8,000 ft) in Colorado and up to 50% of individuals [2] ascending to higher altitudes. It may progress to life-threatening pulmonary and cerebral edema in a minority of individuals. The incidence of HAPE is 1 per 10,000 skiers in Colorado and up to 1 per 100 climbers at 4,300 meters (>14,000 ft). Fig. 2 depicts the development and progression of symptoms as the function of increasing altitude. Sojourners to altitude over 4,000 meters (13,123 ft) have detectable psychomotor, learning and special memory impairment. At extreme altitude above 7,500 meters (24,600 ft) one-third of climbers experience hallucinations. Those who have been above 6,000 meters (19,685 ft) have brain MRI changes in the form of white matter hyperintensities and cortical atrophy [3,4].

The diagnosis of AMS is based on the presence of headache and at least one of the following symptoms: poor appetite, nausea, vomiting, dizziness, fatigue, or sleep disturbance. A Lake Louise Score of 3 or greater out of a maximum score of 15 in the presence of headache denotes AMS (Table 1). There is evidence to suggest that the development of mild cerebral edema due to restricted cerebral venous drainage relative to increased cerebral blood flow in response to hypoxia and intracellular fluid shift is the underlying mechanisms for increased intracranial pressure and the development of high altitude headache and AMS [5]. Because there is so much individual variation in the rate of acclimatization and the risk of AMS, none of the general recommendations offers complete protection against AMS. However, the Himalayan Rescue Association with its more conservative guidelines of ascending no more than 300 meters/day with a rest day for every additional 600-900 meters (2,600 ft) and no single day gain greater than 800 meters will make it less likely to develop AMS [6].

### 1.4 Breathing in High Altitude

At altitudes above 3,000 meters (>9,800 ft) and with arterial oxygen saturation (SaO_2_) <90%, almost all healthy subjects experience periodic breathing during sleep. Periodic breathing at high altitude may also occur in wakefulness, especially during periods of drowsiness [7-9]. The key essential element in inducing periodic breathing is heightened chemosensitivity to hypoxia, involving both primary carotid body chemoreceptor simulations along with secondary effects of enhancing the central chemoreceptor sensitivity to CO_2_ [10,11]. Apnea and periodic breathing during sleep occur within minutes of hypoxic exposure. As shown in the sleeping sojourners to 4,300 meter altitude, hyperventilation in response to the reduced arterial oxygen tension (PaO_2_) occurs first, followed by oscillations in tidal volume and minute ventilation. Ventilatory acclimatization to altitude is the progressive increase in ventilation and and subsequent fall in PaCO_2_ in the order of hours to days and months [12]. Although on balance, periodicity is usually evident early in sleep and during light stages, periodic breathing particularly during NREM sleep can persist despite improvement in sleep architecture [13].

### 1.5 Sleep in High Altitude

**Fig. 3:**
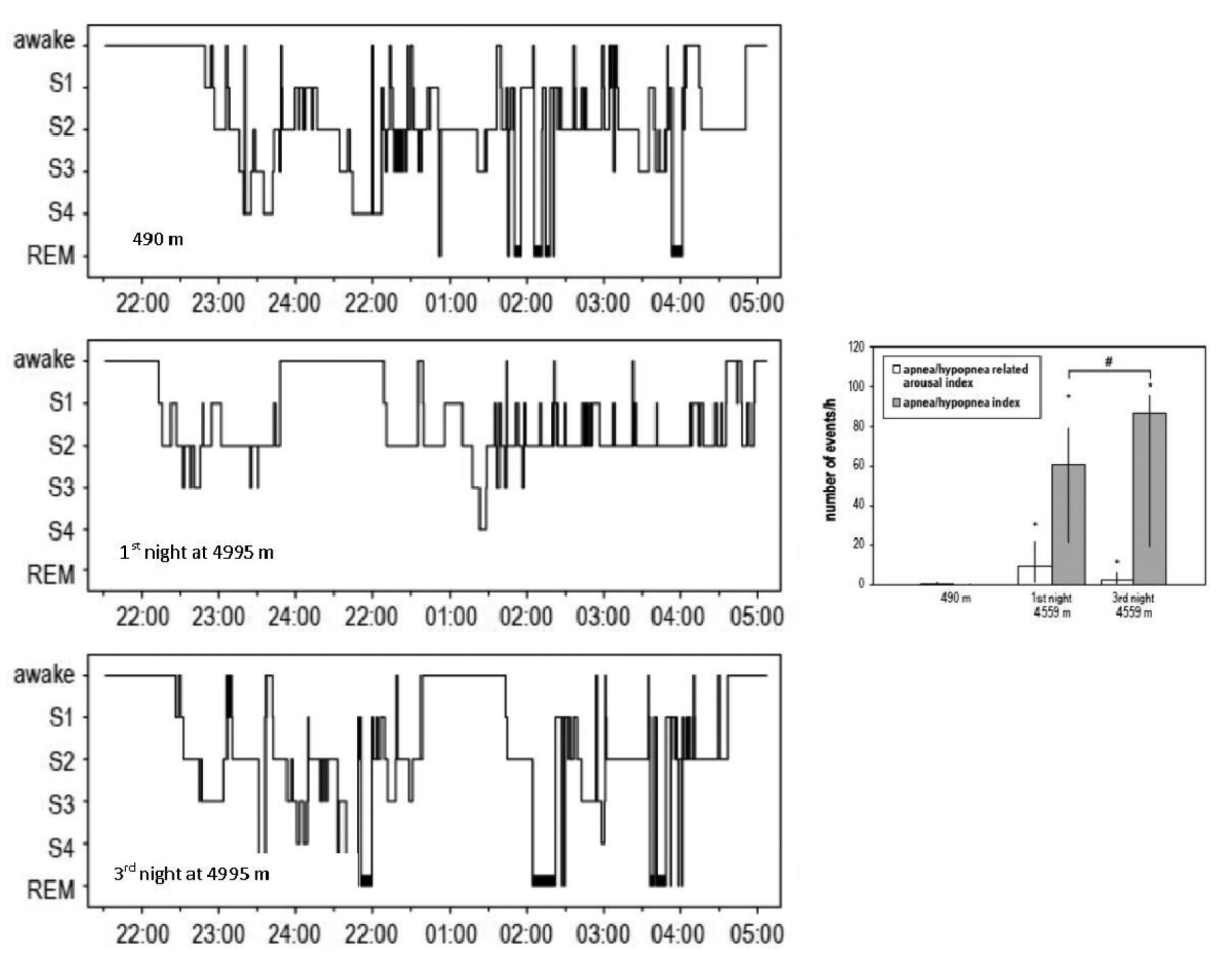
A normal subject, who climbed to Regina Margherita hut at 4995 meters (>16,000 ft) within 24 hours, had a reduction in total sleep time, slow wave sleep, and REM sleep, and an increased number of arousals on polysomnography during the first night at 4995 meters compared to 490 m. Three days of acclimatization resulted in improvement in sleep architecture with increases in slow wave sleep, REM sleep and a reduction in the arousal index despite a further increase in apneas/hypopneas (inset), suggesting that periodic breathing was not the predominant cause of sleep disturbances at altitude

At altitudes over 4,000 meters (>13,000 ft), the duration and efficiency of sleep are reduced, sleep onset time and amount of awake activity are increased and both slow wave sleep and rapid eye movement (REM) sleep are reduced [14]. However, after 3 nights at the same altitude there is improvement in sleep architecture with increases in slow wave sleep, REM sleep and a reduction in the arousal index (Fig. 3) [15]. In the Andes, South America, at an altitude of 4,330 meters (>14,000 ft) healthy natives have sleep duration and distribution of stages comparable to those in subjects at lower altitude [16]. However, these native highlanders have periodic breathing with cyclical oxygen desaturation and elevated hematocrit [17]. Similar observations have been made in Sherpas native to high altitude in Himalaya but not in Sherpas native to low altitude [18].

In summary, during the initial ascent to high altitudes sleep is disrupted and breathing pattern is periodic. However, after few nights at the same altitude sleep quality improves while period breathing persists. The latter is the process of acclimatization to maintain a relatively higher SaO_2_ at high altitude that is also seen in high altitude natives.

## 2. PREVENTION AND TREATMENT

Patients with obstructive (asthma and chronic obstructive pulmonary disease) and restrictive (e.g., interstitial lung disease). diseases as well those with pulmonary vascular disease are at high risk for exacerbation of their respiratory disorder at high altitude regardless of whether they have normal oxygen saturation at sea level [19]. In addition to exposure to hypoxia of high altitude, other atmospheric conditions such as dry and cold air can exacerbate their condition. Frequent use of inhaled bronchodilator prior to the trek and intense physical exertion during the treks increased the likelihood of asthma exacerbation [20]. However, studies of asthmatics at an altitude of 5,050 meters (16,568 ft) showed no worsening of asthma and decreased bronchial hyperresponsiveness, believed to be related to high corticosteroids and catecholamines circulating levels [21]. On the other hand, acute exposure to high altitude can worsen bronchial hyperresponsiveness [22]. Asthma should be under good control and in stable state with adequate privation of medications before the trek to high altitude. Patients with respiratory disorders planning destinations to high altitude should be evaluated by healthcare providers knowledgeable in this area.

The best way to lower the risk of high altitude illnesses is by allowing the body to acclimatize which is a time-dependent process. Aerobic exercise training confers no advantage for acclimatization. In fact, highly fit individuals may increase the risk of developing high altitude illnesses by ascending too rapidly. The Himalayan Rescue Association with its more conservative guidelines of ascending no more than 300 meters/day with a rest day for every additional 600-900 meters (2,600 ft) and no single day gain greater than 800 meters will make it less likely to develop AMS [6]. Staged, gradual ascent to high altitude is an effective way to prevent AMS and blunt sleep-related symptoms, but this may be inconvenient or not possible as in flying to high altitude research facilities or high altitude rescue operations. The treatment and prophylaxis of AMS and sleep abnormalities at high altitude are similar. Pharmacologic approaches include carbonic anhydrase inhibitors and hypnotic agents. Noninvasive positive pressure ventilation or dead-space breathing masks have also been evaluated as a possible treatment [23].

### 2.1 Acetazolamide

Acetazolamide, a carbonic anhydrase inhibitor, is the most common and best studied agent used for amelioration of AMS and sleep disturbance at high altitude [24-27]. For individuals who are susceptible to AMS or for whom a rapid ascent is necessary, acetazolamide is the drug of choice for pharmacologic prevention of AMS [1]. Acetazolamide, unlike other drugs, accelerates the rate of acclimatization rather than masking symptoms by reducing periodic breathing in sleep with improvement in SaO_2_ [28,29]. Acetazolamide markedly improved both the mean level and the stability of arterial oxygenation during sleep and reduced the proportion of sleep time during which periodic breathing occurred [30-32]. Acetazolamide is a respiratory stimulant that causes metabolic acidosis and hyperventilation by increasing renal excretion of bicarbonate [33]. These changes mimic the natural process of acclimatization. An effective prophylactic dose of acetazolamide is 125 mg twice daily to be taken a day before ascent and continued for 2 days after the highest sleeping altitude [31,34]. However, it can speed recovery from AMS if taken after symptoms have developed. Allergic reactions to acetazolamide are uncommon. As a non-antimicrobial sulfonamide, it does not cross-react with antimicrobial sulfonamides. However, it is best avoided by people with history of anaphylaxis to any sulfa. People with history of severe penicillin allergy have occasionally had allergic reactions to acetazolamide. Ibuprofen 600 mg every 8 hours was recently found to help prevent AMS, although it was not as effective as acetazolamide. However, it is an over-the-counter drug, inexpensive, and well tolerated.

### 2.2 Dexamethasone

Dexamethasone is effective for preventing and treating AMS and HACE, and perhaps HAPE as well [35]. Unlike acetazolamide, if the drug is discontinued at altitude before acclimatization, rebound can occur. Acetazolamide is preferable to prevent AMS while ascending, with dexamethasone reserved for treatment, as an adjunct to descent. The adult dose is 4 mg every 6 hours taken orally.

### 2.3 Nifedipine

Nifedipine prevents HAPE and ameliorates it as well [36]. For prevention, it is generally reserved for people with history of HAPE. The adult dose for prevention or treatment is 30 mg of extended release every 12 hours, or 20 mg every 8 hours by mouth. Descent to lower altitude is mandatory.

### 2.4 Benzodiazepines

Several studies suggest the safety and potential utility of benzodiazepines for the treatment of sleep disturbance of high altitude [14,37,38]. Temazepam (7.5 mg or 10 mg) shortened sleep latency, decreased arousals, increased sleep efficiency, increased REM sleep with subjectively better quality sleep in climbers sleeping above 4,000 meters (>13,000 ft) [14,38]. The non-benzodiazepine sedative agents zolpidem (10 mg) and zaleplon (10 mg) have each been found to be effective in improving sleep architecture and consolidation at high altitude. A study of these agents at a simulated altitude of 4,000 meters (>13,000 ft), and another in trekkers at 3,613 meters (12,000 ft), showed that, compared with placebo, both agents improved sleep efficiency and decreased wakefulness, and that zolpidem increased slow wave sleep. Neither drug, however, had an effect on nocturnal respiratory pattern or SaO_2_ nor decreased daytime cognitive or physical performance [39,40].

### 2.5 Other Modalities

Oxygen supplementation improves period breathing by lessening the hypoxic ventilatory drive. However, the issues with logistics of using oxygen at high altitude preclude its routine use except for the treatment of HAPE and high altitude cerebral edema. Promising effects of other modalities to improve high altitude periodic breathing, including non-invasive ventilation and increasing dead space breathing remain to be further investigated in humans. The results of studies of *Ginko biloba* in prevention of AMS have been inconsistent. Acetazolamide is considered far superior prophylaxis for AMS prevention.

## 3. CONCLUSIONS

The sensation of disrupted sleep after ascent to high altitude is associated with frequent awakenings, which in part reflects sleep fragmentation by periodic breathing. Periodic breathing is an abnormal ventilatory pattern in which apneas and hypopneas alternate with periods of hyperventilation. The hypoxic stimulation of the peripheral chemoreceptors in high altitude increases the CO_2_ chemosensitivity. This facilitates the development of central apneas when PaCO_2_ falls below the apnea threshold. Sleep disruption decreases with time at moderate altitude and is also considerably reduced by pretreatment with acetazolamide. Recent studies suggest that benzodiazepines and other hypnotic agents may improve sleep quality without apparent adverse effects [41]. Positive airway pressurization or increased dead space breathing by oronasal mask tends to improve periodic breathing and AMS scores.

## Figures and Tables

**Table 1:** Lake louise score for the diagnosis of Acute Mountain Sickness (AMS)

Headache	No headache		0
	Mild headache		1
	Moderate headache		2
	Severe headache		3

Gastrointestinal symptoms	None		0
	Poor appetite or nausea		1
	Moderate nausea/vomiting		2
	Severe nausea/vomiting		3

Fatigue/weakness	Not tired or weak		0
	Mild fatigue/weakness		1
	Moderate fatigue/weakness		2
	Severe fatigue/weakness		3

Dizziness/lightheadedness	Not dizzy		0
	Mild dizziness		1
	Moderate dizziness		2
	Severe dizziness, incapacitating		3

Difficulty sleeping	Slept as well as usual		0
	Did not sleep as well as usual		1
	Woke many times, poor sleep		2
	Could not sleep at all		3

		Total Score:	

A diagnosis of AMS is based on a total score of 3 or more from the questionnaire; a rise in altitude within the last 4 days; Presence of a headache PLUS; Presence of at least one other symptom
